# The Peripheral Hearing and Central Auditory Processing Skills of Individuals With Subjective Memory Complaints

**DOI:** 10.3389/fnins.2020.00888

**Published:** 2020-08-21

**Authors:** Dona M. P. Jayakody, Holly K. Menegola, Jessica M. Yiannos, Jack Goodman-Simpson, Peter L. Friedland, Kevin Taddei, Simon M. Laws, Michael Weinborn, Ralph N. Martins, Hamid R. Sohrabi

**Affiliations:** ^1^Ear Science Institute Australia, Subiaco, WA, Australia; ^2^Ear Sciences Centre Faculty of Health and Medical Sciences, The University of Western Australia, Crawley, WA, Australia; ^3^School of Human Sciences, The University of Western Australia, Crawley, WA, Australia; ^4^Department of Otolaryngology Head Neck Skull Base Surgery, Sir Charles Gairdner Hospital, Nedlands, WA, Australia; ^5^School of Medicine, University Notre Dame, Fremantle, WA, Australia; ^6^School of Medical and Health Sciences, Edith Cowan University, Joondalup, WA, Australia; ^7^Collaborative Genomics Group, School of Medical and Health Sciences, Edith Cowan University, Joondalup, WA, Australia; ^8^School of Pharmacy and Biomedical Sciences, Faculty of Health Sciences, Curtin Health Innovation Research Institute, Curtin University, Bentley, WA, Australia; ^9^School of Psychological Science, The University of Western Australia, Nedlands, WA, Australia; ^10^Department of Biomedical Sciences, Faculty of Medicine and Health Sciences, Macquarie University, Sydney, NSW, Australia; ^11^Centre for Healthy Ageing, School of Psychology and Exercise Science, Murdoch University, Murdoch, WA, Australia

**Keywords:** hearing loss, central auditory processing, dementia, subjective memory complaints, *APOE*-ε4

## Abstract

**Purpose:**

This study examined the central auditory processing (CAP) assessment results of adults between 45 and 85 years of age with probable pre-clinical Alzheimer’s disease – i.e., individuals with subjective memory complaints (SMCs) as compared to those who were not reporting significant levels of memory complaints (non-SMCs). It was hypothesized that the SMC group would perform significantly poorer on tests of central auditory skills compared to participants with non-SMCs (control group).

**Methods:**

A total of 95 participants were recruited from the larger Western Australia Memory Study and were classified as SMCs (*N* = 61; 20 males and 41 females, mean age 71.47 ±7.18 years) and non-SMCs (*N* = 34; 10 males, 24 females, mean age 68.85 ±7.69 years). All participants completed a peripheral hearing assessment, a CAP assessment battery including Dichotic Digits, Duration Pattern Test, Dichotic Sentence Identification, Synthetic Sentence Identification with Ipsilateral Competing Message (SSI-ICM) and the Quick-Speech-in-Noise, and a cognitive screening assessment.

**Results:**

The SMCs group performed significantly poorer than the control group on SSI-ICM −10 and −20 dB signal-to-noise conditions. No significant differences were found between the two groups on the peripheral hearing threshold measurements and other CAP assessments.

**Conclusions:**

The results suggest that individuals with SMCs perform poorly on specific CAP assessments in comparison to the controls. The poor CAP in SMC individuals may result in a higher cost to their finite pool of cognitive resources. The CAP results provide yet another biomarker that supports the hypothesis that SMCs may be a primary indication of neuropathological changes in the brain. Longitudinal follow up of individuals with SMCs, and decreased CAP abilities should inform whether this group is at higher risk of developing dementia as compared to non-SMCs and those SMC individuals without CAP difficulties.

## Introduction

With over 47 million individuals living with dementia worldwide in 2015, this syndrome is considered a growing global epidemic in older adults (Prince et al., 2015). According to the World Alzheimer Report, the number of people with a dementia diagnosis will rise to 74.7 million in 2030 and 132 million by 2050 (Prince et al., 2015). Alzheimer’s disease (AD) is the most common cause of dementia in older adults accounting for 60–80% of all-cause dementia (Lambert et al., 2014).

The neuropathological changes associated with AD, including the deposition of amyloid plaques and neurofibrillary tangles, start 20–30 years before the clinical diagnosis (Serrano-Pozo et al., 2011). The National Institute of Aging-Alzheimer’s Association Work Groups on diagnostic guidelines suggest that the course of AD can be divided into three subsequent stages: (1) the pre-clinical stage of AD (no impairment in cognition on standard assessments and biomarker evidence for AD), (2) mild cognitive impairment (MCI) due to AD (impairment on memory or other domains of cognition on a standard assessment and biomarker evidence for AD), and (3) dementia due to AD (dementia and biomarker evidence for AD plus subtle cognitive decline) (Jack et al., 2011; McKhann et al., 2011; Sperling et al., 2011; Albert et al., 2013). Current evidence suggests that the self-reported decline in memory or other cognitive functions in the presence of normal performance on neuropsychological measures is associated with an increased risk for future cognitive decline and AD dementia (Glodzik-Sobanska et al., 2007; Wang et al., 2011; Scheef et al., 2012).

With no cure or effective treatment currently insight, it is critical to identify those factors that may prevent or delay cognitive decline and dementia in older adults. Of the potentially modifiable risk factors, untreated hearing loss contributes up to 8% of the modifiable risk factors in mid-life (Livingston et al., 2020). Age-related hearing loss (ARHL) or presbycusis is a multifactorial disorder affecting hearing acuity that varies from mild to profound and results from lifetime insults to the auditory system (Gates and Mills, 2005). Prevalence data indicates that 63% of adults aged 70 years and older have a >25 dBHL speech frequency (4 PTA average of 0.5, 1, 2, and 4 kHz) hearing loss in their better ear (Lin et al., 2011b). Evidence from both cross-sectional (Jayakody et al., 2017) and longitudinal (Lin et al., 2011a; Deal et al., 2016) studies confirmed an increase in the risk of cognitive impairment and incident dementia among older adults with ARHL (Lin et al., 2011a; Deal et al., 2016). In an 11-year longitudinal study, baseline peripheral hearing loss was associated with the increased risk of incident AD (1.27 times per 10 dB hearing loss).

Central auditory processing (CAP) impairment refers to auditory perceptual difficulties that cannot be explained by impairment in peripheral hearing but refers to the impairment in the central auditory pathway affecting speech understanding such as neural transmission, feature extraction and detecting small gaps in the speech which is crucial in speech discrimination, integrating and separating binaural auditory information (Humes et al., 2012; Musiek and Chermak, 2013; Fortunato et al., 2016). A typical hearing complaint of older adults is their inability to understand speech, especially in the presence of background noise (CHABA, 1988). Together, peripheral hearing loss, cognitive decline, aging, and diminished CAP skills (i.e., decline in speech-in-noise processing, dichotic processing and temporal processing skills, or a combination of all these abilities) contribute to the poor speech understanding skills of older adults (Humes et al., 2012; Musiek and Chermak, 2013).

Results from several longitudinal studies suggest that central hearing or CAP skills, in the absence of a severe peripheral hearing loss, are associated with high incidences of cognitive decline and AD dementia (Gates et al., 2002, 2008, 2011) and may precede cognitive impairments and dementia diagnosis by three to 12 years (Gates et al., 1996). These studies have demonstrated that individuals with CAP dysfunction were at a significantly higher risk for incident dementia with hazard ratios ranging from 9.9 (95% CI, 3.6–26.7) to 23.3 (95% CI, 6.6–82.7) (Gates et al., 1996, 2011). Central auditory processing impairment is now considered the primary auditory impairment associated with an increased risk of AD (Panza et al., 2018).

A few studies have previously investigated the CAP skills in individuals diagnosed with MCI (Idrizbegovic et al., 2013; Edwards et al., 2017) and dementia (Gates et al., 2011; Golden et al., 2015; Quaranta et al., 2015). However, to date, no study has investigated the CAP skills in individuals with subjective memory complaints (SMCs). The SMCs include self-reported concerns about one’s memory, which is a major component of subjective cognitive decline (SCD) and is considered a risk factor for future dementia due to AD (Choe et al., 2018). Investigating whether SMC individuals have poor CAP skills compared to those with non-SMCs would provide a unique opportunity to identify people at high risk of developing dementia or who are probably at pre-clinical phases of AD and dementia, years before clinical diagnosis (Jessen et al., 2014; Rönnlund et al., 2015).

The primary aim of our study was to measure the peripheral and central hearing of participants with SMCs and non-SMCs. We hypothesized that participants in the SMC group would perform significantly poorer on tests of peripheral and central hearing assessment compared to non-SMC participants.

Many studies have explored the contribution of genetic factors to ARHL (Karlsson et al., 1997; Gates et al., 1999; Friedman et al., 2009), and to AD (Poorkaj et al., 1998; Cai et al., 2015; Hardy, 2017). However, there is no conclusive evidence on whether cognitive impairment/dementia and ARHL share a common genetic etiology. Kurniawan et al. (2012) reported that apolipoprotein E (*APOE*) ε4 allele, a major genetic risk factor for AD, is strongly associated with poorer hearing thresholds, whereas, Mener et al. (2016) reported that participants with two *APOE* ε4 alleles had better hearing thresholds at 1.0 kHz (β = −8.56, *p* = 0.021) compared to those with no *APOE* ε4 alleles. However, whether *APOE* ε4 allele status is associated with impaired peripheral hearing sensitivity and CAP skills is yet to be investigated. Therefore, we also investigated the association between peripheral and central auditory functions and cognitive functions, and the impact of *APOE* ε4 carriage as the primary genetic risk factor for late-onset AD. We hypothesized that the *APOE* ε4 carriers would perform significantly poorer than the non-carries on central and peripheral hearing assessments.

## Methods

### Study Design: Prospective Study

Participants were recruited from the Western Australia Memory Study (WAMS), a longitudinal study into aging and dementia. The ethics approval for this study was received from the Ramsay Health Care WA| SA Human Research Ethics Committee (previously, the Hollywood Private Hospital Ethics Committee, Western Australia). All procedures were undertaken per this approval. The participants completed an informed consent form prior to taking part in the study. The hearing assessments were conducted by a trained researcher under the supervision of a qualified audiologist, and the cognitive assessments were performed by a qualified psychometric rater trained and certified by the study neuropsychologists.

### Participants

A total of 95 individuals who have been speaking Australian English for 10 years or longer, aged between 45–85 years, in a general state of good health and with a 4PTA (average of better ear hearing thresholds at 0.5, 1, 2, and 4 kHz) of less than 40 dBHL were recruited for the study. None of the participants used hearing aids. All participants were recruited from the WAMS (Sohrabi et al., 2012).

## Materials and Procedure

The assessment materials included measures of cognition, psychological status, and peripheral and central hearing.

Demographic information: All participants completed a demographic questionnaire that was asking for information on age, sex, education, and family history of stroke, dementia, diabetes, smoking, and high blood pressure.

### Hearing Assessment

The hearing assessment consisted of two components: peripheral and central.

#### Peripheral Hearing Assessment

The hearing assessment included an otoscopic examination and pure-tone audiometric testing. The pure-tone audiometric assessment was conducted using a KUDUwave 5000, Type 2 clinical audiometer (Emoyo, Johannesburg, South Africa). Bilateral air-conduction thresholds between 0.5 and 8 kHz and bone-conduction thresholds between 0.5 and 4 kHz were obtained through standard audiometric assessment in a sound-attenuating booth that meets the standard for audiometric testing. This task took approximately 20 min to complete.

#### Central Auditory Processing Assessment

The CAP functions were assessed using a battery of five central auditory tests to identify the central auditory deficits. The assessments were presented at 65 dBHL, for participants who had 4PTA > 25 dBHL. For those who had 4PTA < 25 dBHL, test materials were presented at 4PTA + 20 dB.

The following CAP tests were administered.

##### Dichotic Digits Test

Dichotic Digits Test (DDT; Musiek et al., 1991) is a test of binaural integration. The participants were instructed that “you will hear two numbers in each of your ears. Listen carefully and repeat all the numbers you hear. The order doesn’t matter. If you are unsure of the numbers, please guess.” The participant was presented with one practice presentation and 25 assessed presentations. Each digit repeated back was marked as correct or incorrect. The ears were scored separately, and the order in which the participant repeated the digits back did not matter. Scores above 90% in each ear are considered within the normal limits for adults with normal hearing sensitivity and under the age of 65 years. For adults over the age of 65 years or with a hearing loss, the cut-off is 82% (Musiek, 1983).

##### Dichotic Sentence Identification

Dichotic Sentence Identification (DSI; Fifer et al., 1983) is a test of binaural separation. During the DSI, the participant was presented with 20 sentence pairs from a printed list of six sentences. In each sentence pair, the participant was presented with one of the six sentences in each ear; the sentences always differed in each ear. The 20 sentence pairs were presented in two blocks of 10; in the first block, the participant was asked to attend to the sentence being presented to their right ear and then indicate the sentence number that corresponded to the sentence presented in their directed ear. In the second block, the participants were asked to attend to their left ear and complete the same task. Each trial was marked as correct or incorrect and then each ear was scored as a percentage correct.

##### Duration Pattern Test

In the Duration Pattern Test (DPT; Musiek, 1994), participants were presented with 1 kHz tones in sequences of three, where each tone could have a duration of either 250 ms (short) or 500 ms (long) with 300 ms intervals between the tones in the sequence of three tones (Musiek and Chermak, 1994). The participants were asked to linguistically label each of the tone durations as either short (S) or long (L) in each series, for example, “short, long, long.” There were six possible combinations of the three tones (SSL, LLS, LSL, SLS, LSS, SLL). Each three-item sequence of the tones had to be identified with the lengths in the correct order to be scored as correct. The participant was presented with three practice presentations and then with 30 assessed presentations, with 15 in each ear. Percent correct scores for each ear were recorded.

##### Synthetic Sentence Identification with Ipsilateral Competing Message

In the Synthetic Sentence Identification with Ipsilateral Competing Message (SSI-ICM; Speaks et al., 1967; Orchik and Burgess, 1977) test, the participant selected which nonsense sentence out of 10 was being presented against a competing narrative in the same ear. The competing narrative was a passage about Davy Crockett presented by the same presenter, ipsilateral to the target sentence (Jerger and Jerger, 1974; Supplementary Material 1). Studies have shown both hearing loss and age can affect SSI-ICM scores (Gates et al., 2008), therefore, the lists were presented at 65 dBHL for those who had 4PTA > 25 dBHL. For those who had 4PTA < 25 dBHL, test materials were presented at 4PTA + 20 dB. Four sets of ten sentences were presented at + 10 dB speech-to-noise ratio (SNR), 0 dB SNR, −10 dB SNR, and −20 dB SNR conditions. The target sentences presented at 10 dB above the competing narrative in the + 10 dB SNR condition, 10dB below the competing narrative in the −10dB condition and 20 dB below the competing narrative in the −20 dB condition. The target sentence and competing narrative were presented at the same level in the 0 dB SNR condition.

Percent correct score was calculated for each test condition and each ear separately.

##### Quick Speech-in-Noise

In the Quick Speech-in-Noise (QuickSIN; Killion et al., 2004) test, the participant was presented with two practice sentences and two pre-recorded test lists in each ear; each list is comprised of six sentences with five target words in each. The multi-talker babble increased by 5 dB per sentence between 40 and 65 dBHL, creating varying conditions between a 25 dB speech-to-noise ratio and a 0 dB SNR for the final sentence. The participant was asked to repeat the sentence back to the assessor and was scored one point for each word they correctly repeated. The total score was then subtracted from 25.5 to calculate the signal-to-noise ratio loss (Killion et al., 2004). Examples are provided in Supplementary Material 2.

### Cognitive Assessment

The WAMS is a longitudinal study of community-dwelling older adults in Perth Western Australia, specifically enriched for recruiting individuals with SMCs. All participants complete a comprehensive neuropsychological assessment, including self and informant scales and questionnaires. The WAMS assessment battery takes up to 3 h and has various measures assessing global cognition, verbal and visual episodic memory, attention, executive functions, language, orientation, visuospatial skill, and so on. In addition, the participants provide blood samples and undergo brain imaging. Self-reports and informant-reports surveys and questioners are collected for the psychological, cognitive, and personality characteristics of the WAMS participants. As this paper is focused on the relationship between hearing loss and SMCs, we have not reported on the neuropsychological measures. Further information on the neuropsychological measures used as part of WAMS can be found in Sohrabi et al. (2019).

To assess the participants’ general cognitive abilities and screen for potential cognitive impairment, the WAMS utilizes the Montreal Cognitive Assessment (MoCA) (Nasreddine et al., 2005). Only participants with a MoCA cut-off score of ≥23 were considered cognitively normal and recruited for this study (Rossetti et al., 2011). For those participants with borderline or concerning results, the full neurocognitive battery, as well as the clinical data, are examined by the study Lead Neuropsychs (HRS and MW) to ensure that such results are appropriately communicated with participants and their medical doctors. Of note, the endpoint for participation in the WAMS is a diagnosis of MCI, AD, or other types of dementia.

In this study, participants were divided into two groups: a control (non-SMC), and a high-risk group (SMC) based on completing a self-reported survey, namely the Memory Assessment Clinics Questionnaire (MAC-Q; Crook et al., 1992). The MAC-Q is a self-report questionnaire designed to capture concerns about memory decline. The first five questions assess age-related decline in memory on daily activities and the last question asks about overall memory decline. Participants in the current study were asked to rate all six items on a 5-point Likert scale ranging from “Much better now” to “Much poorer now,” compared to when they were at high school or college. The total score on the MAC-Q ranges from 7 to 35, and higher scores indicate more complaints about one’s memory abilities. Participants were considered SMCs if they scored ≥25 on the MAC-Q (Crook et al., 1992).

### Genotyping

In this study, the *APOE* ε4 allele was determined using TaqMan^®^ genotyping assays (rs7412, assay ID: C ____904973_10; rs429358, assay ID: C___3084793_20) from DNA extracted from 5ml of a whole blood sample using QIAamp DNA Blood Maxi Kits (Qiagen, Hilden, Germany). TaqMan^®^ genotyping assays were performed on a QuantStudio 12K Flex^TM^ Real-Time-PCR systems (Applied Biosystems, Foster City, CA) using the TaqMan^®^ GTXpress^TM^ Master Mix (Life Technologies) as per manufacturer instructions. *APOE* ε4 carriers included participants with at least one ε4 allele (i.e., ε2/ε4, ε3/ε4, ε4/ε4) and non-carriers included no copies of the ε4 allele (ε2/ε2, ε2/ε3, ε3/ε3).

### Statistical Analysis

Data were recorded in Excel and statistically analyzed using IBM SPSS Statistics, Version 25.0 (IBM Corp, Armonk, NY). Analyses of Co-Variance (ANCOVA) were run for each ear in each test to test the hypotheses that SMC participants perform significantly poorer than non-SMC participants in tests of peripheral and central hearing. Age was considered a covariate and adjustments for multiple comparisons were made using Bonferroni corrections. The participants of this study cohort had a wide age range and (50–85 years). Also, age is the primary risk factor for both ARHL and cognitive impairment in older adults (Glisky, 2007). In addition, SMCs increases in frequency (number of times given individual complaints) and prevalence as a factor of age (Koivisto et al., 1995; Coley et al., 2008). Therefore, adjusting for age was required to be able to disentangle the effect of age on the relationship between hearing loss and SMCs. Assumptions for linearity, homogeneity and homoscedasticity were met for each test and there were no outliers in the data, as assessed by no cases with standardized residuals greater than ±3 standard deviations. Data are reported as unadjusted mean ± standard deviation unless otherwise stated.

To investigate the relationship between SMC and age, gender, better ear 4PTA and CAP tests, we have conducted binomial logistic regression with the presence of SMCs as the dependent variables and age, gender, better ear 4PTA and CAP tests as independent variables.

## Results

Demographic details of the participants are reported in Table 1. Of the 95 participants who were selected for the study, 61 participants (20 males and 41 females, mean age 71.47 ±7.18 years) had MAC-Q scores ≥25, hence, categorized as SMC. Thirty-four participants (10 males, 24 females, mean age 68.85 ±7.69 years) had MAC-Q scores < 25, hence categorized as non-SMC. Mean and SD values for both SMC and non-SMC groups for all the CAP tests are reported in Table 2. There was no significant interaural asymmetry observed in any of the participants. CAPS test protocols suggest assessing each ear separately and the scores were calculated for each ear as well. Hence, rather than averaging scores of right and left ear, we analyzed results for each ear which is a routine way of analyzing hearing assessment results.

**TABLE 1:** Demographic data of the participants.

**Parameters**	**Total *N* (%)/Mean (SD)**
**Sex**	
Female	65 (68.4%)
Male	30 (31.6%)
Apolipoprotein E (*APOE*) ε4 carriers	26 (27.4%)
Subjective memory complainers (SMCs)	61 (64.1%)

**TABLE 2:** Mean and standard deviation scores of central auditory processing tests for both participant groups.

**Task**	**SMC**			**Non-SMC**		
	**N**	**Mean**	**SD**	**N**	**Mean**	**SD**
Age	61	71.47	7.18	34	68.85	7.70
Years of Formal Education	61	13.59	2.62	34	14.74	2.80
Right Ear 4PTA (0.5,1,2, and 4 kHz average) dBHL	59	25.57	15.02	34	22.72	12.24
Left Ear 4PTA (0.5,1,2, and 4 kHz average) dBHL	59	25.41	12.85	34	23.09	10.74
Better Ear 4PTA (0.5,1,2, and 4 kHz average) dBHL	59	23.01	12.25	34	19.93	9.87
Right ear 2 HF PTA average (6 and 8 kHz) dBHL	59	33.90	21.19	34	30.93	20.27
Left Ear 2 HF PTA average (6 and 8 kHz) dBHL	59	37.77	19.75	34	35.59	19.46
Better ear 2 HF PTA average (6 and 8 kHz) dBHL	59	31.13	19.68	34	28.24	19.32
Dichotic Digits Test (DDT) Right Ear-Percent correct score	59	86.07	13.52	33	85.08	14.37
Dichotic Digits Test (DDT) Left Ear-Percent correct score	59	80.83	13.45	33	81.65	13.39
Quick Speech in Noise Test (QuickSIN) Right Ear-dBSNR	60	5.96	4.45	33	5.43	4.01
Quick Speech in Noise Test (QuickSIN) Left Ear-dBSNR	60	5.91	4.49	33	5.69	4.45
Duration Pattern Test (DPT) Right Ear-Percent correct score	60	89.44	18.33	33	95.33	7.79
Duration Pattern Test (DPT)- Left Ear-Percent correct score	60	90.29	17.43	33	93.52	13.61
Dichotic Speech Identification Test-Right Ear-Percent correct score	60	97.33	7.78	33	97.77	5.71
Dichotic Speech Identification Test-Left Ear-Percent correct score	60	93.22	16.86	32	96.02	6.76
Synthetic Sentence Identification-Ipsilateral Competing Message (SSI-ICM) Right Ear−20 dBSNR condition- Percent correct score	56	23.21	21.67	33	42.12	19.33
Synthetic Sentence Identification-Ipsilateral Competing Message (SSI-ICM) Left Ear−20 dBSNR condition- Percent correct score	57	29.47	24.16	33	53.91	21.14
Synthetic Sentence Identification-Ipsilateral Competing Message (SSI-ICM) Right Ear−10 dBSNR condition- Percent correct score	58	53.97	22.08	33	67.58	17.32
Synthetic Sentence Identification-Ipsilateral Competing Message (SSI-ICM) Left Ear−10 dBSNR condition- Percent correct score	58	54.14	21.36	33	68.18	17.22
Synthetic Sentence Identification-Ipsilateral Competing Message (SSI-ICM) Right Ear 0 dBSNR condition-Percent correct score	59	87.93	17.30	33	90.61	13.91
Synthetic Sentence Identification-Ipsilateral Competing Message (SSI-ICM) Left Ear 0 dBSNR condition-Percent correct score	59	93.05	13.93	33	94.24	10.01
Synthetic Sentence Identification-Ipsilateral Competing Message (SSI-ICM) Right Ear + 10 dBSNR condition-Percent correct score	59	98.47	8.05	34	99.41	2.39
Synthetic Sentence Identification-Ipsilateral Competing Message (SSI-ICM) Left Ear + 10 dBSNR condition-Percent correct score	59	98.14	8.20	34	99.71	1.72
The Montreal Cognitive Assessment(MOCA) Total Score – out of 30	61	26.75	2.08	34	26.56	2.58
Memory Complaint Questionnaire (MAC-Q) Total Score-out of 35	61	27.33	3.03	34	23.50	3.83

Analysis of Co-variance results of the SMC and non-SMC participants revealed no significant difference between peripheral left and right ear 4PTA and 2 HF PTA (high frequency average of 6 and 8 kHz) of the SMC and non-SMC participant groups (Table 3). Both groups of participants had their 4PTA mean hearing thresholds within normal limits (<25 dBHL). The SMC group performed significantly poorer than the non-SMC group on SSI-ICM −10 dB SNR [right ear, *F* (1, 89) = 6.59, *p* = 0.012; left ear *F* (1, 89) = 7.87, *p* = 0.006) and −20 dB SNR (right ear *F* (1, 87) = 13.85, *p* < 0.001; and left ear *F* (1,88) = 20.06, *p* < 0.001] conditions. No significant differences were found between participant groups for the other CAP measures i.e., Quick-SIN, DDT, DSI, and DPT tasks (Table 3).

**TABLE 3:** Analysis of Co-variance results of the subjective memory complainers and non-memory complainers.

**Parameter**	***F***	***P* value**	**Partial η^2^**	**Observed power**
Better ear 4PTA average (0.5,1,2, and 4 kHz)	*F* (1,91) = 0.60	0.43	0	0.12
Right ear 4PTA average (0.5,1,2, and 4 kHz)	*F* (1,91) = 0.34	0.56	0	0.08
Left ear 4 PTAaverage (0.5,1,2, and 4 kHz)	*F* (1,91) = 0.16	0.69	0	0.06
Better ear 2 HF PTA average (6 and 8 kHz)	*F* (1,91) = 0.09	0.75	0	0.06
Right ear 2 HF PTA average (6 and 8 kHz)	*F* (1,91) = 0.14	0.7	0	0.06
Left ear 2 HF PTA average (6 and 8 kHz)	*F* (1,91) = 0.00	0.97	0	0.05
Dichotic Digits Test (DDT) Right Ear	*F* (1,90) = 1.45	0.23	0.01	0.22
Dichotic Digits Test (DDT) Left Ear	*F* (1,90) = 2.82	0.59	0	0.08
Duration Pattern Test (DPT) Right Ear	*F* (1,91) = 3.40	0.06	0.03	0.44
Duration Pattern Test (DPT) Left Ear	*F* (1,91) = 0.99	0.32	0.01	0.16
QuickSpeech in Noise Test (QuickSin) Right Ear	*F* (1,91) = 0.01	0.9	0.01	0.05
QuickSpeech in Noise Test (QuickSin) Left Ear	*F* (1,91) = 0.38	0.53	0	0.09
Dichotic Speech Identification Test (DSI) Right Ear	*F* (1,91) = 0.01	0.89	0	0.05
Dichotic Speech Identification Test (DSI) Left Ear	*F* (1,90) = 0.10	0.75	0	0.06
Synthetic Sentence Identification-Ipsilateral Competing Message (SSI-ICM) −20 dBSNR Right Ear	*F* (1,87) = 13.85	0.001*	0.13	0.95
Synthetic Sentence Identification-Ipsilateral Competing Message (SSI-ICM) −20 dBSNR Left Ear	*F* (1,88) = 20.06	0.001*	0.19	0.99
Synthetic Sentence Identification-Ipsilateral Competing Message (SSI-ICM) −10 dBSNR Right Ear	*F* (1,89) = 6.59	0.012*	0.06	0.71
Synthetic Sentence Identification-Ipsilateral Competing Message (SSI-ICM) −10 dBSNR Left Ear	*F* (1,89) = 7.87	0.006*	0.08	0.79
Synthetic Sentence Identification-Ipsilateral Competing Message (SSI-ICM) 0 dBSNR Right Ear	*F* (1,90) = 0.00	0.97	0	0.05
Synthetic Sentence Identification-Ipsilateral Competing Message (SSI-ICM) 0 dBSNR Left Ear	*F* (1,90) = 0.01	0.91	0	0.05
Synthetic Sentence Identification-Ipsilateral Competing Message (SSI-ICM) + 10 dBSNR Right Ear	*F* (1,91) = 0.02	0.87	0.02	0.05
Synthetic Sentence Identification-Ipsilateral Competing Message (SSI-ICM) + 10 dBSNR Left Ear	*F* (1,91) = 0.38	0.53	0	0.09

*P* < 0.05 marked with an asterisk (*). Age has been considered as a co-variate in the analysis.

Results of the binomial logistic regression failed to reveal any significant association between 4PTA and SMC. However, SSI-ICM −20 and −10 dB SNR conditions were statistically significant. For SSI-ICM left ear −20dB SNR condition the model explained 24.8% of the total variance, sensitivity was 79.6% and the specificity was 57.6% and the positive predictive value was 75.43%. For SSI-ICM right ear −20 dB SNR condition the model explained 33.1% of the total variance, sensitivity was 74.5% and the specificity was 54.5% and the positive predictive value was 73.21%. Results of the binomial logistic regression analysis are reported in Supplementary Table 3.

### AD Genetic Risk Factor and Hearing

Of the 95 participants tested 26 were *APOE* ε4 carriers (10 males, 16 females; mean age 68.85 ±8.33 years), and 69 were non-carriers (20 males, 49 females; mean age 71.17 ±7.03 years). No significant difference was observed between carriers and non-carriers on better and poorer ear 4PTA scores or better and poorer ear 2HF PTA average. The performance of carriers and non-carriers on all central auditory test measures were not significantly different (Table 4). Of the total cohort, 20 were SMC and positive for *APOE* ε4 carriage. However, when compared with the remaining 75 participants, there was no significant difference between the two groups on any of the hearing tests.

**TABLE 4:** Analysis of Co-variance results for the *APOE* e4 carriers and non-carriers.

**Parameter**	***F***	***P* value**	**Partial η^2^**	**Observed power**
Better ear 4PTA average (0.5,1,2, and 4 kHz)	*F* (1,91) = 0.22	0.63	0.00	0.02
Poor ear 4PTA average (0.5,1,2, and 4 kHz)	*F* (1,91) = 0.38	0.53	0.00	0.09
Right ear 4PTA average (0.5,1,2, and 4 kHz)	*F* (1,91) = 0.73	0.39	0.00	0.13
Left ear 4 PTA average (0.5,1,2, and 4 kHz)	*F* (1,91) = 0.02	0.87	0.00	0.05
Dichotic Digits Test (DDT) Right Ear	*F* (1,90) = 0.55	0.48	0.01	0.11
Dichotic Digits Test (DDT) Left Ear	*F* (1,90) = 3.92	0.51	0.04	0.50
Duration Pattern Test (DPT) Right Ear	*F* (1,91) = 0.35	0.55	0.00	0.09
Duration Pattern Test (DPT) left ear	*F* (1,91) = 0.17	0.89	0.00	0.05
QuickSpeech in Noise Test (QuickSin) Right Ear	*F* (1,91) = 0.29	0.59	0.00	0.08
QuickSpeech in Noise Test (QuickSin) Left Ear	*F* (1,91) = 0.06	0.80	0.00	0.05
Dichotic Speech Identification Test (DSI) Right Ear	*F* (1,91) = 0.34	0.56	0.00	0.08
Dichotic Speech Identification Test (DSI) Left Ear	*F* (1,91) = 1.54	0.21	0.01	0.23
Synthetic Sentence Identification-Ipsilateral Competing Message (SSI-ICM) −20 dBSNR Right Ear	*F* (1,87) = 1.05	0.31	0.01	0.17
Synthetic Sentence Identification-Ipsilateral Competing Message (SSI-ICM) −20 dBSNR Left Ear	*F* (1,88) = 0.00	0.94	0.00	0.05
Synthetic Sentence Identification-Ipsilateral Competing Message (SSI-ICM) −10 dBSNR Right Ear	*F* (1,89) = 1.13	0.28	0.01	0.14
Synthetic Sentence Identification-Ipsilateral Competing Message (SSI-ICM) −10 dBSNR Left Ear	*F* (1,89) = 0.03	0.85	0.00	0.05
Synthetic Sentence Identification-Ipsilateral Competing Message (SSI-ICM) 0 dBSNR Right Ear	*F* (1,90) = 0.26	0.61	0.00	0.08
Synthetic Sentence Identification-Ipsilateral Competing Message (SSI-ICM) 0 dBSNR Left Ear	*F* (1,90) = 0.20	0.65	0.00	0.07
Synthetic Sentence Identification-Ipsilateral Competing Message (SSI-ICM) + 10 dBSNR Right Ear	*F* (1,91) = 0.00	0.92	0.00	0.05
Synthetic Sentence Identification-Ipsilateral Competing Message (SSI-ICM) + 10 dBSNR Left Ear	*F* (1,91) = 0.13	0.71	0.00	0.06

*Age has been considered a co-variate in this analysis.*

## Discussion

A significant difference between the SMC and non-SMC groups was observed on tests of SSI-ICM −10 and −20 dB SNR conditions. Of note, mean values of right and left ear 4PTA results of both groups were not significantly different. The hearing thresholds of left ear, right ear, and better ear 4PTA for both groups were approximately 25 dB. Therefore, we conclude that the SSI-ICM results were not influenced by the peripheral hearing loss.

Central auditory processing difficulties are known to place additional demands and strain on an individual’s cognitive processing capacity (Bellis and Jorgensen, 2014). This is due to the requirement for additional “upstream” resources to facilitate auditory perception and other higher-order central auditory processes (Bellis and Jorgensen, 2014). This study’s findings suggest that while there may be a decline in some CAP skills in individuals with SMCs, this deficit may only become visible when the CAP system is significantly challenged, and cognitive resources are exhausted or depleted, such as in the SSI-ICM −10 or −20 SNR subtests. Moreover, speech processing is one of the most challenging and cognitively demanding CAP tasks that rely on several auditory processing skills, including auditory attention, temporal resolution, temporal patterning, and auditory closure (Chermak and Lee, 2005). Speech processing becomes even more difficult and cognitively complex when the speech signal is distorted by other competing messages, meaning apt auditory attention is also required to discriminate and accurately understand what is being said (Gates et al., 2010).

The SSI-ICM test appears to be sensitive to the decline of CAP skills in MCI populations and accurately predicts the future clinical diagnosis of AD (Gates et al., 2008 and 2010; Edwards et al., 2017). Gates et al. (2008) found that individuals with a diagnosis of MCI performed significantly poorer on the 0 dB SNR condition of the SSI-ICM compared to a control group. The −10 and −20 dB SNR conditions of the SSI-ICM were not assessed in the Gates et al. (2008) study. However, given the known progression of the neuropathology and differences in cognition between MCI and SMC groups, it can be assumed that a more difficult task, where the signal is more distorted and therefore requires more cognitive resources, is necessary to differentiate between SMC and control groups as compared to other groups.

To understand why the SMC group performed poorly in the SSI-ICM task, we need to understand the nature of this task. The human auditory system perceptually segregates the competing acoustic stimuli into different streams in a process known as auditory scene analysis (Bregman, 1990). The auditory scene analysis makes use of the spatial, spectral and supra-segmental composition, as well as temporal information to segregate the foreground from background information (Bregman, 1990). It does this by matching the incoming acoustic information to previously stored auditory “templates,” mediated by attentional mechanisms (Carlyon et al., 2001; Billig et al., 2013). Synthetic Sentence Identification with Ipsilateral Competing Message is a test of auditory closure that helps fill in the missing information in the presence of a competing signal (Speaks et al., 1967). Synthetic sentences are third-order approximations of English sentences. These sentences contain seven semantically meaningless words. They are designed to reduce the listener’s reliance on linguistic skills while preserving the syntax and temporal features of the English language (Jerger and Jerger, 1974). The SSI-ICM test sentences were presented at different signal to noise ratios in the same ear making the task more challenging at higher signal-to-noise ratios. The −10 and −20 dB SNR condition of the SSI-ICM was considered the most challenging test of speech processing used. The competing message was 10 dB louder than the target signal/sentence in −10 dB SNR, and 20 dB louder than the target signal in −20 dB SNR conditions, which results in a more distorted signal in comparison to the Quick-SIN and 0 and +10 dB SNR conditions of the SSI-ICM.

Consequently, the SMC group performed significantly poorer than the control group only in a subset of CAP tests, despite there being no significant difference between the peripheral hearing thresholds of the two groups. This is not surprising, as a more distorted speech signal requires greater processing for speech understanding, which then subsequently places increased strain on the higher-order auditory processing skills and consequently requires more cognitive resources (Bellis and Jorgensen, 2014). To recognize speech in challenging situations, proficient listeners rely either on high predictability or clarity of the speech (Bradlow and Alexander, 2007). The SSI-ICM target sentences are semantically and syntactically unrelated; hence predictability is poor. Medwetsky (1994, 2011), reported that the spatial location of the sound sources and fundamental frequency differences in talkers allows the speakers to segregate the acoustic signals into meaningful units. In the absence of both spatial and fundamental frequency difference cues, short sentences with 5–6 word length can still be recalled to a certain extent (Medwetsky, 1994). In this study SSI-ICM sentences contained seven words in each sentence list, hence the listeners could not rely on spatial cues or talker gender differences to segregate the information into different acoustic streams, making the task extremely difficult.

Several neural networks are required to work synchronously to facilitate speech recognition. For example, the lateral prefrontal cortex is involved in the organization and execution of behavior, speech and reasoning (Fuster, 2001), the posterior portion of Broca’s area plays a vital role in processing phonological working memory information (Gelfand and Bookheimer, 2003), primary and associated auditory cortices with stream segregation during auditory scene analysis (Deike et al., 2004, 2010; Gutschalk et al., 2007), posterior superior temporal gyrus and planum temporale with the detailed perceptual representation of segregated objects (Schönwiesner et al., 2007), parietal cortex in mapping sensory inputs to motor actions (Cusack, 2005; Cohen, 2009) and mid-ventrolateral prefrontal cortex driving the top-down attentional processes (Schönwiesner et al., 2007).

Tuwaig et al. (2017) reported an association between poor SSI-ICM scores (−10 dB SNR) and atrophy in the right Heschl’s gyrus cortical thickness and thinner right parahippocampal and entorhinal cortices, bilateral precuneus, occipital cortex, left inferior parietal lobule, inferior and mid temporal gyri on structural magnetic resonance imaging (MRI), after adjusting for age, gender, education, pure-tone hearing, and *APOE* ε4 status. Heschl’s gyrus is involved in the temporal processing of the auditory signal (Zatorre and Belin, 2001). The inferior parietal lobe plays a vital role in auditory working memory: integration of sensory-motor information and monitoring and updating the location of the sound source (Alain et al., 2008). Changes in the cortical thickness of inferior parietal cortices are observed early in the neurodegeneration from normal to MCI, and neurodegeneration of parahippocampal/entorhinal regions is seen in progression from MCI to AD (Devanand et al., 2012). Neuroimaging studies that examined the biomarkers of early AD (accumulation of global Aβ and tau burdens) in SMC individuals have found an increased accumulation of early tauopathy in the entorhinal cortex (Buckley et al., 2017) as well as an increase in global Aβ burden (Rodda et al., 2010; Amariglio et al., 2012); both are considered neuropathological hallmarks of AD (Braak and Braak, 1997). Attending to a challenging task such as SSI-ICM requires the optimal functioning of neural networks. Hence, we propose that neuropathological impairment associated with SMC may have impeded their ability in segregating foreground from background information under higher signal-to-noise ratios reflecting in poor performances in the SSI-ICM task.

Furthermore, if numerous cognitive processes and auditory processing skills are required for the SSI-ICM, this suggests the task is highly taxing on an individual’s finite pool of cognitive resources. In this study perhaps individuals with SMC could compensate for impaired CAP skills by drawing on more upstream cognitive resources until the task became too demanding and the cognitive resources are exhausted, creating significant differences between the SMCs and control group. Therefore, given the numerous cortical areas and cognitive processes involved in attending to the SSI-ICM task, it can be expected that this task would be sensitive to the effects of potential neurodegeneration in SMC populations.

In our study, the SMC group did not perform significantly poorer than the non-SMCs in the peripheral hearing assessment. Both participant groups performed within the normal range for the global cognitive functions, yet SMCs performed poorly in some of the CAP functions. Two prominent theories have been proposed to explain the interaction between peripheral hearing and CAP in older adults: the central effects of biological aging (CEBA) and central effects of peripheral pathology (CEPP; Willott, 1996). Central effects of biological aging hypothesis is that changes in central hearing occur due to the anatomical and physiological changes that occur in the central nervous system as a result of aging without any peripheral deficits. Evidence from animal studies that have reported age-related pathophysiological changes in the central auditory pathway without any peripheral impairment (Walton et al., 1998; Ouda and Syka, 2012) seems to support this hypothesis. The CEPP hypothesis argues that the pathophysiological changes in the central auditory pathway occur as a result of peripheral hearing impairment (Willott, 1996). Increase in the cognitive load resulting from peripheral hearing loss (CHABA, 1988; Lindenberger and Baltes, 1994) or longstanding hearing loss could lead to impaired cognitive functions (CHABA, 1988; Lindenberger and Baltes, 1994; Schneider and Pichora-Fuller, 2000; Lin et al., 2011b, 2013; Humes et al., 2012). As there was no significant difference between the peripheral hearing thresholds of the SMC and non-SMC groups, our study supports the CEBA theory; therefore, we conclude that CAP deficits that we have seen may indicate early signs of neurodegeneration.

Similar to previous findings (O’Grady et al., 2007; Tuwaig et al., 2017), we also failed to observe any significant difference between *APOE* ε4 carriers and non-carriers on peripheral hearing thresholds. However, in the Leiden 85-plus Study (Kurniawan et al., 2012), participants with the *APOE* ε4/ε4 genotype had more than moderately-severe levels of hearing loss (*n* = 6; mean 56.1 dBHL) and participants with the *APOE* ε3/ε4 or ε2/ε4 genotype showed moderate levels of (*n* = 89, mean 51.0 dBHL) while those without the *APOE*-ε4 allele (*n* = 340) had the poorest levels of hearing loss (48.9 dBHL). In our study, the recruited participants had 4PTA [4-frequency pure-tone average 500, 1000, 2000, and 4000 Hz) hearing thresholds within approximately 25 dBHL but were significantly younger than the Kurniawan et al. (2012) study, furthermore, the number of carriers was not large enough to confirm whether such differences would exist in higher levels of hearing losses. However, the mechanism underlying these observations should be explored in a larger sample size.

This study was considered a cross-sectional pilot study and consequently was limited in its interpretation and relatively small sample size. Ideally, the CAP skills of more participants should be examined longitudinally to note any deterioration in participants’ cognition and/or CAP skills. Our study also did not have access to brain imaging that could have been informative in substantiating the presence or absence of neurodegenerative processes (if any) in their earlier stages.

In summary, the results of this study are cautiously promising and have established a potential diagnostic opportunity in identifying probable pre-clinical AD that may assist in delaying or arresting the cognitive decline in older adults. However, these results should be validated and replicated in larger groups over a more extended period. Furthermore, future research can combine these two measures (CAP and SMC) to examine the predictive value of each one as well as the increased accuracy when they are used together. While SSI and SMC are independent, the added benefit of using these two in identifying individuals at higher risk of dementia is another potential research that can inform the field.

## Data Availability Statement

The datasets presented in this article are not readily available due to Australian privacy laws. Requests to access the datasets should be directed to DJ (dona.jayakody@earscience.org.au).

## Ethics Statement

The studies involving human participants were reviewed and approved by Hollywood Private Hospital Ethics Committee, Western Australia (HPH-139). The patients/participants provided their written informed consent to participate in this study.

## Author Contributions

DJ: study design and manuscript preparation. HM, JG-S, and JY: hearing data collection. KT, SL, and MW: genetic assessment. RM and HS: cognitive assessment. All authors provided interpretation of the results and reviewed the manuscript.

## Conflict of Interest

HS has received ruminations for working with Pfizer and Takeda. HS research is partially supported by the Australian Alzheimer’s Research Foundation.

The remaining authors declare that the research was conducted in the absence of any commercial or financial relationships that could be construed as a potential conflict of interest.
