# Supplementary material for: The Peripheral Hearing and Central Auditory Processing Skills of Individuals With Subjective Memory Complaints
Source: Front Neurosci. 2020 Aug 21;14:888. doi: 10.3389/fnins.2020.00888 (PMC7475691; doi:10.3389/fnins.2020.00888)
Supplement: Supplementary file 1 [file Data_Sheet_1.docx]

**Supplementary material 1: Synthetic Sentence Identification with Ipsilateral Competing Message (SSI-ICM)**

1. Small boat with a picture has become

2. Built the government with the force almost

3. Go change your car colour is red

4. Forward march said the boy had a

5. March around without a care in your

6. The neighbour who said business is better

7. Battle cry and be better than ever

8. Down by the time is real enough

9. Agree with him only to find out

10. Women view men with green paper should

**Supplementary material 2: Quick Speech in Noise (Quick-SIN)**

**Presentation Level:** _______dB

**Norms:**

**PRACTICE – Track 6 – List 4 – First two sentences only**

| List No. | Sentence |
| --- | --- |
| 1 | The sense of smell is better than that of touch |
| 2 | He picked up the dice for a second roll |

**Track 7 - List 5 Ear:__________________**

|  | Sentence | S/N | Score |
| --- | --- | --- | --- |
| 1 | To have is better than to wait and hope | 25 |  |
| 2 | The screen before the fire kept in the sparks | 20 |  |
| 3 | Thick glasses helped him read the print | 15 |  |
| 4 | The chair looked strong but had no bottom | 10 |  |
| 5 | They told wild tales to frighten him | 5 |  |
| 6 | The force equal to that would move the earth | 0 |  |

25.5- TOTAL= _______SNR Loss Total:________

**Track 11- List 9 Ear:__________________**

|  | Sentence | S/N | Score |
| --- | --- | --- | --- |
| 1 | Take shelter in this tent, but keep still | 25 |  |
| 2 | The little tales they tell are false | 20 |  |
| 3 | Press the pedal with your left foot | 15 |  |
| 4 | The black trunk fell from the landing | 10 |  |
| 5 | Cheap clothes are flashy but don’t last | 5 |  |
| 6 | At night the alarm roused him from a deep sleep | 0 |  |

25.5- TOTAL= _______SNR Loss Total:________

**Track 13- List 11 Ear:__________________**

|  | Sentence | S/N | Score |
| --- | --- | --- | --- |
| 1 | The marsh will freeze when cold enough | 25 |  |
| 2 | A gray mare walked before the colt. | 20 |  |
| 3 | Bottles hold four kinds of rum | 15 |  |
| 4 | He wheeled the bike past the winding road. | 10 |  |
| 5 | Throw out the used paper cup and plate. | 5 |  |
| 6 | The wall phone rang loud and often. | 0 |  |

25.5- TOTAL= _______SNR Loss Total:________

**Track 14 – List 12 Ear:__________________**

|  | Sentence | S/N | Score |
| --- | --- | --- | --- |
| 1 | The hinge on the door creaked with old age | 25 |  |
| 2 | The bright lanterns were gay on the dark lawn | 20 |  |
| 3 | He offered proof in the form of a large chart | 15 |  |
| 4 | Their eyelids droop for want of sleep | 10 |  |
| 5 | There are many ways to do these things | 5 |  |
| 6 | We like to see clear weather | 0 |  |

25.5- TOTAL= _______SNR Loss Total:________

TOTAL SCORE LEFT: __________ TOTAL SCORE RIGHT: __________

**Table 3.** Binomial logistic regression model with age, gender, years of education, BE 4PTA and central auditory processing tests as co-variates.

|  | B | S.E. | Wald | df | Sig. | Odds ratio | 95% C.I. for Odds ratio | |  |
| --- | --- | --- | --- | --- | --- | --- | --- | --- | --- |
|  |  |  |  |  |  |  | Lower | Upper |  |
| Age | 0.06 | 0.04 | 2.28 | 1.00 | 0.13 | 1.06 | 0.98 | 1.14 |  |
| Gender | -0.32 | 0.55 | 0.35 | 1.00 | 0.56 | 0.72 | 0.25 | 2.12 |  |
| Education years | -0.15 | 0.09 | 2.71 | 1.00 | 0.10 | 0.86 | 0.71 | 1.03 |  |
| BE 4PTA | 0.01 | 0.02 | 0.22 | 1.00 | 0.64 | 1.01 | 0.97 | 1.06 |  |
| Dichotic Digits Right ear | 0.03 | 0.02 | 2.35 | 1.00 | 0.13 | 1.03 | 0.99 | 1.07 |  |
|  | | | | | | | | |  |
| Age | 0.05 | 0.04 | 1.48 | 1.00 | 0.22 | 1.05 | 0.97 | 1.12 |  |
| Gender | -0.33 | 0.53 | 0.38 | 1.00 | 0.54 | 0.72 | 0.25 | 2.05 |  |
| Education (years) | -0.14 | 0.09 | 2.25 | 1.00 | 0.13 | 0.87 | 0.73 | 1.04 |  |
| BE 4PTA | 0.01 | 0.02 | 0.11 | 1.00 | 0.74 | 1.01 | 0.96 | 1.06 |  |
| Dichotic Digits Left ear | 0.01 | 0.02 | 0.45 | 1.00 | 0.50 | 1.01 | 0.98 | 1.05 |  |
|  | | | | | | | | |  |
| Age | 0.04 | 0.04 | 1.27 | 1.00 | 0.26 | 1.04 | 0.97 | 1.11 |  |
| Gender | -0.23 | 0.55 | 0.18 | 1.00 | 0.67 | 0.79 | 0.27 | 2.34 |  |
| Education (years) | -0.15 | 0.09 | 2.62 | 1.00 | 0.11 | 0.86 | 0.72 | 1.03 |  |
| BE 4PTA | 0.02 | 0.03 | 0.31 | 1.00 | 0.58 | 1.02 | 0.96 | 1.07 |  |
| QuickSIN Right ear | -0.05 | 0.07 | 0.43 | 1.00 | 0.51 | 0.96 | 0.83 | 1.10 |  |
|  | | | | | | | | |  |
| Age | 0.05 | 0.04 | 2.02 | 1.00 | 0.16 | 1.05 | 0.98 | 1.13 |  |
| Gender | -0.24 | 0.54 | 0.20 | 1.00 | 0.65 | 0.78 | 0.27 | 2.27 |  |
| Education (years) | -0.18 | 0.10 | 3.52 | 1.00 | 0.06 | 0.84 | 0.70 | 1.01 |  |
| BE 4PTA | 0.03 | 0.03 | 1.18 | 1.00 | 0.28 | 1.03 | 0.98 | 1.09 |  |
| QuickSIN Left ear | -0.12 | 0.07 | 2.80 | 1.00 | 0.09 | 0.88 | 0.76 | 1.02 |  |
|  |  |  |  |  |  |  |  |  |  |
| Age | 0.04 | 0.03 | 1.37 | 1.00 | 0.24 | 1.04 | 0.97 | 1.11 |  |
| Gender | -0.47 | 0.55 | 0.75 | 1.00 | 0.39 | 0.62 | 0.21 | 1.82 |  |
| Education (years) | -0.13 | 0.09 | 1.86 | 1.00 | 0.17 | 0.88 | 0.74 | 1.06 |  |
| BE 4PTA | 0.01 | 0.02 | 0.04 | 1.00 | 0.84 | 1.01 | 0.96 | 1.05 |  |
| Duration Pattern Test- Right ear | -0.04 | 0.02 | 2.55 | 1.00 | 0.11 | 0.97 | 0.92 | 1.01 |  |
|  |  |  |  |  |  |  |  |  |  |
| Age | 0.04 | 0.03 | 1.18 | 1.00 | 0.28 | 1.04 | 0.97 | 1.11 |  |
| Gender | -0.37 | 0.54 | 0.46 | 1.00 | 0.50 | 0.69 | 0.24 | 1.99 |  |
| Education (years) | -0.14 | 0.09 | 2.22 | 1.00 | 0.14 | 0.87 | 0.73 | 1.04 |  |
| BE 4PTA | 0.01 | 0.02 | 0.05 | 1.00 | 0.82 | 1.01 | 0.96 | 1.05 |  |
| Duration Pattern Test- Left ear | -0.01 | 0.02 | 0.72 | 1.00 | 0.40 | 0.99 | 0.96 | 1.02 |  |
|  |  |  |  |  |  |  |  |  |  |
| Age | 0.03 | 0.03 | 0.98 | 1.00 | 0.32 | 1.03 | 0.97 | 1.11 |  |
| Gender | -0.34 | 0.55 | 0.38 | 1.00 | 0.54 | 0.72 | 0.25 | 2.09 |  |
| Education (years) | -0.14 | 0.09 | 2.50 | 1.00 | 0.11 | 0.87 | 0.72 | 1.04 |  |
| BE 4PTA | 0.01 | 0.02 | 0.05 | 1.00 | 0.82 | 1.01 | 0.96 | 1.05 |  |
| Dichotic Speech Identification- Right ear | -0.01 | 0.04 | 0.02 | 1.00 | 0.88 | 1.00 | 0.93 | 1.07 |  |
|  |  |  |  |  |  |  |  |  |  |
| Age | 0.03 | 0.04 | 0.77 | 1.00 | 0.38 | 1.03 | 0.96 | 1.11 |  |
| Gender | -0.25 | 0.54 | 0.21 | 1.00 | 0.65 | 0.78 | 0.27 | 2.26 |  |
| Education (years) | -0.13 | 0.09 | 2.09 | 1.00 | 0.15 | 0.88 | 0.73 | 1.05 |  |
| BE 4PTA | 0.01 | 0.02 | 0.07 | 1.00 | 0.79 | 1.01 | 0.96 | 1.05 |  |
| Dichotic Speech Identification- Left ear | -0.01 | 0.02 | 0.07 | 1.00 | 0.80 | 1.00 | 0.96 | 1.04 |  |
|  |  |  |  |  |  |  |  |  |  |
| Age | 0.02 | 0.04 | 0.15 | 1.00 | 0.70 | 1.02 | 0.94 | 1.09 |  |
| Gender | -0.39 | 0.57 | 0.45 | 1.00 | 0.50 | 0.68 | 0.22 | 2.09 |  |
| Education (years) | -0.16 | 0.10 | 2.52 | 1.00 | 0.11 | 0.85 | 0.70 | 1.04 |  |
| BE 4PTA | -0.01 | 0.03 | 0.07 | 1.00 | 0.79 | 0.99 | 0.94 | 1.05 |  |
| SSI-ICM Right ear -20 dB SNR | -0.04 | 0.01 | 10.83 | 1.00 | 0.001* | 0.96 | 0.94 | 0.98 |  |
|  | | | | | | | | |  |
| Age | -0.02 | 0.04 | 0.16 | 1.00 | 0.69 | 0.98 | 0.91 | 1.06 |  |
| Gender | -0.31 | 0.62 | 0.25 | 1.00 | 0.62 | 0.73 | 0.22 | 2.45 |  |
| Education (years) | -0.19 | 0.10 | 3.38 | 1.00 | 0.07 | 0.83 | 0.68 | 1.01 |  |
| BE 4PTA | 0.01 | 0.03 | 0.07 | 1.00 | 0.79 | 1.01 | 0.95 | 1.07 |  |
| SSI-ICM Left ear -20 dB SNR | -0.05 | 0.01 | 14.32 | 1.00 | 0.000* | 0.95 | 0.93 | 0.98 |  |
|  |  |  |  |  |  |  |  |  |  |
| Age | 0.01 | 0.04 | 0.13 | 1.00 | 0.72 | 1.01 | 0.94 | 1.09 |  |
| Gender | -0.40 | 0.55 | 0.53 | 1.00 | 0.47 | 0.67 | 0.23 | 1.98 |  |
| Education (years) | -0.15 | 0.09 | 2.52 | 1.00 | 0.11 | 0.86 | 0.72 | 1.04 |  |
| BE 4PTA | -0.02 | 0.03 | 0.48 | 1.00 | 0.49 | 0.98 | 0.93 | 1.03 |  |
| SSI-ICM -Right ear -10 dB SNR | -0.03 | 0.01 | 5.59 | 1.00 | 0.018* | 0.97 | 0.94 | 0.99 |  |
|  |  |  |  |  |  |  |  |  |  |
| Age | 0.00 | 0.04 | 0.00 | 1.00 | 0.99 | 1.00 | 0.93 | 1.08 |  |
| Gender | -0.92 | 0.60 | 2.33 | 1.00 | 0.13 | 0.40 | 0.12 | 1.30 |  |
| Education (years) | -0.18 | 0.10 | 3.67 | 1.00 | 0.06 | 0.83 | 0.69 | 1.00 |  |
| BE 4PTA | -0.03 | 0.03 | 1.03 | 1.00 | 0.31 | 0.97 | 0.92 | 1.03 |  |
| SSI-ICM- Left ear -10 dB SNR | -0.03 | 0.02 | 5.21 | 1.00 | 0.02* | 0.96 | 0.93 | 0.99 |  |
|  |  |  |  |  |  |  |  |  |  |
| Age | 0.04 | 0.04 | 0.94 | 1.00 | 0.33 | 1.04 | 0.97 | 1.11 |  |
| Gender | -0.39 | 0.56 | 0.49 | 1.00 | 0.49 | 0.68 | 0.23 | 2.03 |  |
| Education (years) | -0.15 | 0.09 | 2.66 | 1.00 | 0.10 | 0.86 | 0.72 | 1.03 |  |
| BE 4PTA | 0.00 | 0.02 | 0.02 | 1.00 | 0.90 | 1.00 | 0.96 | 1.05 |  |
| SSI-ICM - Right ear 0 dB SNR | -0.00 | 0.02 | 0.01 | 1.00 | 0.92 | 1.00 | 0.96 | 1.04 |  |
|  |  |  |  |  |  |  |  |  |  |
| Age | 0.04 | 0.03 | 1.19 | 1.00 | 0.28 | 1.04 | 0.97 | 1.11 |  |
| Gender | -0.36 | 0.54 | 0.45 | 1.00 | 0.50 | 0.70 | 0.24 | 2.01 |  |
| Education (years) | -0.15 | 0.09 | 2.68 | 1.00 | 0.10 | 0.86 | 0.72 | 1.03 |  |
| BE 4PTA | 0.01 | 0.02 | 0.05 | 1.00 | 0.83 | 1.01 | 0.96 | 1.05 |  |
| SSI-ICM - Left ear 0 dB SNR | 0.01 | 0.02 | 0.05 | 1.00 | 0.82 | 1.01 | 0.96 | 1.05 |  |
|  |  |  |  |  |  |  |  |  |  |
| Age | 0.04 | 0.03 | 1.10 | 1.00 | 0.29 | 1.04 | 0.97 | 1.11 |  |
| Gender | -0.44 | 0.54 | 0.68 | 1.00 | 0.41 | 0.64 | 0.22 | 1.84 |  |
| Education (years) | -0.16 | 0.09 | 2.98 | 1.00 | 0.08 | 0.86 | 0.72 | 1.02 |  |
| BE 4PTA | 0.00 | 0.02 | 0.00 | 1.00 | 0.95 | 1.00 | 0.96 | 1.05 |  |
| SSI-ICM - Right ear 10 dB SNR | -0.01 | 0.05 | 0.01 | 1.00 | 0.91 | 0.99 | 0.90 | 1.09 |  |
|  |  |  |  |  |  |  |  |  |  |
| Age | 0.03 | 0.03 | 0.78 | 1.00 | 0.38 | 1.03 | 0.96 | 1.10 |  |
| Gender | -0.46 | 0.54 | 0.72 | 1.00 | 0.40 | 0.63 | 0.22 | 1.83 |  |
| Education (years) | -0.16 | 0.09 | 2.97 | 1.00 | 0.09 | 0.86 | 0.72 | 1.02 |  |
| BE 4PTA | 0.00 | 0.02 | 0.00 | 1.00 | 0.95 | 1.00 | 0.95 | 1.05 |  |
| SSI-ICM -Left ear 10 dB SNR | -0.09 | 0.11 | 0.69 | 1.00 | 0.41 | 0.91 | 0.73 | 1.14 |  |
|  |  |  |  |  |  |  |  |  |  |

P< 0.05 marked with an asterisk (*)
